# Combined inhibition of C5 and CD14 efficiently attenuated the inflammatory response in a porcine model of meningococcal sepsis

**DOI:** 10.1186/s40560-017-0217-0

**Published:** 2017-02-27

**Authors:** Bernt C. Hellerud, Hilde L. Orrem, Knut Dybwik, Søren E. Pischke, Andreas Baratt-Due, Albert Castellheim, Hilde Fure, Grethe Bergseth, Dorte Christiansen, Miles A. Nunn, Terje Espevik, Corinna Lau, Petter Brandtzæg, Erik W. Nielsen, Tom E. Mollnes

**Affiliations:** 1 Department of Immunology, Oslo University Hospital Rikshospitalet, and K.G. Jebsen IRC, University of Oslo, N-0027 Oslo, Norway; 2 Department of Pediatrics, Oslo University Hospital Ullevål and University of Oslo, Oslo, Norway; 3 Department of Anesthesiology, Nordland Hospital and Nord University, Bodø, Norway; 4 Department of Anesthesiology and Intensive Care Unit, Institution of Clinical Science, Sahlgrenska Academy, University of Gothenburg, Gothenburg, Sweden; 5 Research Laboratory, Nordland Hospital, Bodø, Norway; 6 Akari Therapeutics Plc, London, UK; 7 Centre of Molecular Inflammation Research and Department of Cancer Research and Molecular Medicine, Norwegian University of Science and Technology, Trondheim, Norway; 8 Institute of Clinical Medicine, Faculty of Medicine, University of Oslo, Oslo, Norway; 9 Faculty of Health Sciences, K.G. Jebsen TREC, University of Tromsø, Tromsø, Norway

**Keywords:** Endotoxin, Chemokines, Complement, Cytokines, Immune response, *Neisseria meningitidis*, Septic shock, Toll-like receptor

## Abstract

**Background:**

Fulminant meningococcal sepsis, characterized by overwhelming innate immune activation, mostly affects young people and causes high mortality. This study aimed to investigate the effect of targeting two key molecules of innate immunity, complement component C5, and co-receptor CD14 in the Toll-like receptor system, on the inflammatory response in meningococcal sepsis.

**Methods:**

Meningococcal sepsis was simulated by continuous intravenous infusion of an escalating dose of heat-inactivated *Neisseria meningitidis* administered over 3 h. The piglets were randomized, blinded to the investigators, to a positive control group (*n* = 12) receiving saline and to an interventional group (*n* = 12) receiving a recombinant anti-CD14 monoclonal antibody together with the C5 inhibitor coversin.

**Results:**

A substantial increase in plasma complement activation in the untreated group was completely abolished in the treatment group (*p* = 0.006). The following inflammatory mediators were substantially reduced in plasma in the treatment group: Interferon-γ by 75% (*p* = 0.0001), tumor necrosis factor by 50% (*p* = 0.01), Interleukin (IL)-8 by 50% (*p* = 0.03), IL-10 by 40% (*p* = 0.04), IL-12p40 by 50% (*p* = 0.03), and granulocyte CD11b (CR3) expression by 20% (*p* = 0.01).

**Conclusion:**

Inhibition of C5 and CD14 may be beneficial in attenuating the detrimental effects of complement activation and modulating the cytokine storm in patients with fulminant meningococcal sepsis.

## Background

Fulminant meningococcal sepsis is a rapid and devastating infection caused by *Neisseria meningitidis*, characterized by whole-body inflammation and severe disturbances in homeostasis leading to high mortality despite optimal antimicrobial and intensive care treatment [1–3]. Within 12–24 h after onset of the first symptoms, the number of meningococci in the circulation may reach levels as high as 10^8^/mL in the plasma [3]. A massive and complex inflammatory response is triggered, which in turn is harmful to the body and leads to multi-organ failure [1–3]. Treatment-resistant septic shock caused by profound vasodilation and declining cardiac function is the principal cause of death [1–3]. Activation of TLR4-MD2 by lipopolysaccharide (LPS) is considered the most important inflammatory mechanism in meningococcal sepsis [4, 5]. However, different clinical trials aiming to reduce the inflammatory response caused by LPS have failed to improve the outcome in patients with severe sepsis. This includes studies attempting to neutralize LPS or attenuate the response to LPS by blocking different steps of the inflammatory mechanisms, including binding of LPS to TLR4-MD2 or blocking the effect of individual inflammatory mediators like IL-1β and tumor necrosis factor (TNF) [6–9].

Molecular structures of meningococci other than LPS activate different parts of the innate immune system, including the complement system and additional TLRs beside TLR4-MD2 [10]. CD14 serves as a co-factor for several of the TLRs, including TLR4 and TLR2 [11, 12]. Complement is a plasma cascade system with diverse inflammatory effects, driven mainly by activation products which react with leukocyte surface receptors [13, 14]. It can be activated through three initial routes, namely the classical, lectin, and alternative pathway. Activation of all three initial pathways merges and leads to activation of the common complement factors C3 and C5. Further, activation of C5 leads to formation of C5a and the terminal C5b-9 complex (TCC) [15]. C5a is a potent anaphylatoxin with diverse inflammatory effects including stimulation of cytokine production, upregulation of adhesion molecules, paralysis of neutrophils, and increased vascular permeability [16]. TCC incorporates into bacterial lipid membranes as the membrane attack complex and may cause lysis of pathogens, in particular *Neisseria* species. TCC may also be formed in the fluid phase as a soluble form (sC5b-9) and then serves as a valuable marker of complement activation.

We have hypothesized that a combined inhibition of complement and CD14 will efficiently attenuate the inflammatory response in various clinical situations including sepsis [17]. Previously, we demonstrated that upstream inhibition of the inflammatory response by combined blocking of CD14 and complement strongly attenuates a wide range of inflammatory responses induced by meningococci in an ex vivo whole blood model [10]. A reinforced inhibitory effect on a broad spectrum of inflammatory mediators by the combined inhibition has recently also been proven in porcine *E. coli* induced sepsis [18] and mouse and pig polymicrobial sepsis where survival was also increased [19, 20].

The aim of the present study was to investigate the effect of blocking both C5 and CD14 on the inflammatory response induced in vivo in a porcine model of meningococcal sepsis. The model has been developed by our group as the first large animal model simulating meningococcal sepsis and has been proven to be a valuable tool to study inflammatory mechanisms of the disease and potential interventions [5, 21, 22].

## Methods

### Interventional drugs

For inhibition of porcine CD14, a recombinant variant of a well-established anti-porcine CD14 mouse monoclonal IgG2b antibody (Mil2) was used. This novel anti-porcine CD14 antibody (rMIL2) was recently constructed by our group as a mouse-human chimeric, IgG2/IgG4 hybrid antibody keeping the antigen binding site the same as for Mil2 [23]. Thus, rMIL2 exerts comparable ex vivo and in vivo CD14 binding and inhibition efficacies as the original murine clone, demonstrated by competitive binding tests and cytokine release assays. The original Mil2 clone was used in our initial intervention studies of porcine sepsis [18, 24–26]. The inflammatory response was successfully inhibited, but this clone was hampered by undesired effect or functions mediated by the IgG2b Fc part of Mil2 and consequently induced an anaphylactoid-like reaction. In contrast, the IgG2/4 rMIL2 was completely inert with respect to such functions [22] and successfully used in a subsequent study [20] and in the present one. Endotoxin-free recombinant OmCI (coversin), a 16.8-kDa protein, was provided by Akari Therapeutics Plc (London, UK) [27].

### Bacteria

The international reference strain *N. meningitidis* 44/76 (H44/76) was obtained from the National Institute of Public Health, Oslo, Norway. It is characterized as B:15:P1.7,16:L3,7,9 and was originally isolated from a female patient with lethal meningococcal septic shock [28]. Meningococci were grown overnight on Columbia agar and resuspended in sterile PBS. For safety reasons, bacteria were heat inactivated at 57 °C for 30 min, and then frozen at −70 °C until used.

### Animals and experimental groups

Piglets (*Sus scrofa domesticus*, outbred stock) of either sex, weighing 6 kg (range 5.5–6.5 kg) were used. All piglets were clinically healthy and thus none were excluded from the study. The piglets were randomized into two groups, each consisting of 12 animals: an intervention and a positive control group. In addition, two sham control piglets that received saline instead of bacteria were included as reference. The groups were blinded for the investigators. The number of piglets in each group was decided according to previous analysis and experience showing that at least 10 animals in each group were needed to demonstrate differences [5, 18].

### Anesthesia and surgery

The piglets were pre-medicated with 2–3 mg intranasal midazolam at the animal housing facility before transport to laboratory. At the laboratory, venous access was established in one of the ears and thereafter 10–15 mg/kg ketamine was given intravenously (i.v.) before oral intubation using a 4.9-mm inner diameter tube with cuff. After intubation, an i.v. infusion of propofol (10 mg/kg/h) and fentanyl (50 μg/kg/h) was started and mechanical ventilation was established. A 5 F pulmonary artery catheter (Baxter Edwards Laboratories, Irvine, CA) was inserted via the left external jugular vein and guided into a distal pulmonary artery by pressure wave-form analysis. A cannula was inserted in the left carotid artery for continuous recording of arterial pressure and intermittent blood sampling. A urinary catheter was inserted into the urinary bladder via a cystotomy. After the completion of surgery and insertion of catheters, the animals were placed on their right side, remaining in this position for the rest of the experiment.

### Experimental design

After induction of anesthesia and surgery, the intervention group received first a bolus of coversin (1 mg/kg) and thereafter a bolus of rMIL2 (5 mg/kg), administered about 10 min before induction of sepsis. Excess coversin from the bolus not bound to C5 is rapidly removed from circulation [27]. Therefore, to bind C5 synthesized by piglets during the experiment, coversin was infused at 0.2 mg/h throughout the experimental course. Sepsis was induced by i.v. infusion of increasing numbers of *N. meningitidis*. Septic shock was obtained according to the Third International Consensus Definitions for Sepsis and Septic shock, as vasopressors were needed to keep the blood pressure above 65 mmHg [29]. Due to the influence of vasopressors, cardiovascular parameters could not be included as scientific readouts. There was no difference between the positive control group and the intervention group regarding the amount of vasopressors needed to keep the blood pressure above 65 mmHg.

To limit the need for inhibitors, the size of the pigs used in the present study was reduced from about 30-kg to about 6-kg piglets compared to previously published studies employing the porcine model of meningococcal sepsis [5, 21]. However, we found that in these piglets a state of fulminant sepsis was reached within a shorter time than in the larger animals. Thus, the observational period was shortened from 4 h in the previous studies to 3 h in the present study.

Each piglet received a total of 8.4 × 10^9^ bacteria/kg. The positive control group received the same volume of saline as the intervention group. The sham animals also received the same volume of saline. Blood samples were drawn before i.v. infusion of intervention agents (Tbasis), before start of bacterial infusion (T0), and 60, 120, and 180 min after induction of sepsis (T60, T120, and T180). All animals received the same background infusion of Ringer’s acetate, i.e., 10 mL/kg/h during surgery and until 60 min after induction of sepsis, and thereafter 20 mL/kg/h to 120 min and 30 mL/kg/h to 180 min after induction of sepsis. In addition, 10% glucose was given i.v. at 5 mL/kg/h throughout the experimental course to avoid hypoglycemia. Inotrop and vasopressor therapy were given when needed as a therapy for severe and lethal hypotension.

### Functional complement activity

A commercially available enzyme immune assay (Complement system Screen WIESLAB®; Euro Diagnostica, Malmö, Sweden) was used to test functional activity of the classical complement pathway. The kit detects human complement activity, but has been shown to cross-react efficiently with pig [30]. Samples were analyzed in serum, prepared after 1-h clotting, centrifuged, and immediately aliquoted and stored at −70 °C.

### Complement activation

Soluble TCC (sC5b-9) was measured using multiplex xMAP technology (Bio-Plex® Multiplex System, Bio-Rad Laboratories, Inc. Hercules, CA) as previously described [18, 31]. Samples were analyzed in EDTA-plasma prepared immediately after blood sampling. Whole blood was placed on crushed ice, centrifuged within 30 min at 1500 *g* at 4 °C for 20 min, immediately aliquoted, and stored at −70 °C.

### Cytokines

The cytokines TNF, IL-1β, IL-4, IL-6, IL-8, IL-10, and IL-12p40 and Interferons (INF)-α and INF-γ were analyzed in EDTA-plasma obtained as described for soluble TCC above, using multiplex technology (ProcartaPlex, eBioscience, Bender MedSystems GMbH, Vienna, Austria).

### Leukocyte activation

Leukocyte activation was measured by flow cytometry. In pig whole blood, neutrophils are clearly discriminated from mononuclear cells, but lymphocytes and monocytes cannot be separated by forward/side scatter dot plots. For measurement of wCD11R3 on neutrophils (the pig ortholog to human CD11b), blood was collected at Tbasis and T180, anticoagulated with EDTA, and stained with mouse anti-porcine wCD11R3 FITC clone 2 F4/11 or the isotype-matched mouse IgG1-FITC negative control Ab (AbD Serotec, Oxford, UK). The samples were incubated for 15 min in the dark, and red cells were lysed with 0.16 M NH_4_Cl/10 mM NaHCO_3_-/0.12 mM EDTA (Tritiplex III) for 8–10 min in the dark at ambient temperature. Samples were centrifuged at 300 *g* for 5 min at 4 °C, and the cells were washed with PBS, 0.1% BSA (Biotest, Dreieich, Germany). The samples were then resuspended in PBS, 0.1% BSA before flow cytometry (FACSCalibur; Becton Dickinson, Franklin Lakes, NJ). Values are given in mean fluorescence intensity (MFI).

### Data presentation and statistical analysis

Statistical analyses were performed with GraphPad Prism version 6 (GraphPad Software, San Diego, CA). The positive control group was compared with the treatment group using two-way ANOVA. Area under the curve (AUC) was used as a single measure of the total difference between groups in the amount of each cytokine secreted.

The difference in wCD11R3 upregulation on granulocytes in the intervention group and the positive control group was measured at only one time-point (T180) in addition to baseline and was analyzed by Student’s *t* test. For all statistical analyses, *p* < 0.05 was considered statistically significant. Two sham animals were included as references.

## Results

### Complement activation

**Fig. 1:**
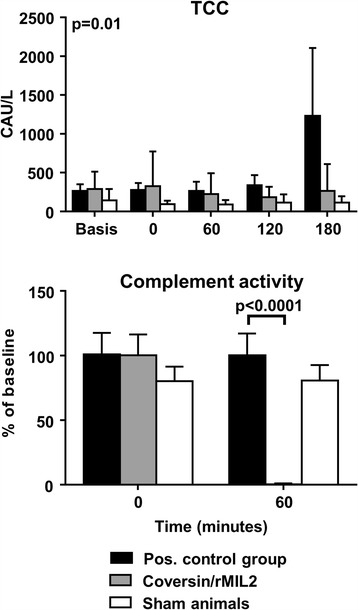
*Upper panel:* Complement activation was measured as plasma TCC by multiplex technology at different time points during the experiments (mean with 95% CI). The positive control group and the group treated with coversin and rMIL2 (coversin/rMIL2) contained 12 animals each. Two sham animals are shown for comparison. *Lower panel:* Complement activity of the classical pathway was measured at different time points by the Complement system Screen WIESLAB® assay (mean with 95% CI). Statistical significance is given for the difference between the positive control and the treatment group

TCC was used as a measure of complement activation at the different time points during the experiment. In the positive control group, TCC increased from mean 273 (±87; 95% CI) complement arbitrary units (CAU)/L at T0 to mean 1233 (±871) CAU/L at T180 (*p* = 0.01) (Fig. 1, upper panel), confirming systemic activation of complement. No increase of TCC was seen in the treatment group. Complete inhibition of complement activity in the treatment group after administration of coversin was further confirmed at T60 by the classical pathway WIESLAB® assay, which detects the total complement activity of the classical and terminal pathway (Fig. 1, lower panel).

### Cyotokines

#### *IFN-γ:*

**Fig. 2:**
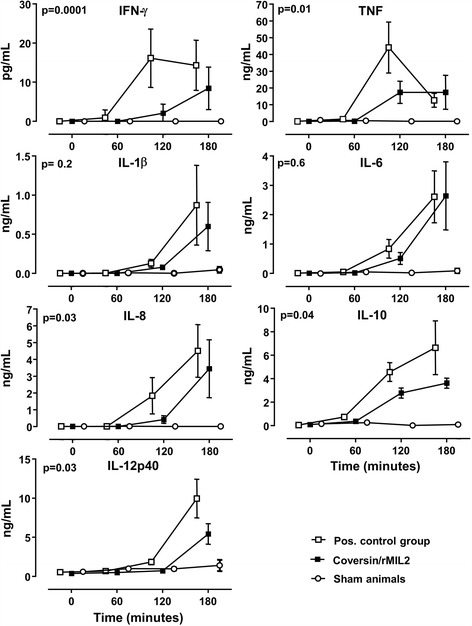
Plasma cytokines (INF-γ, TNF, IL-1β, IL-6, IL-8, IL-10, and IL12p40) were measured by multiplex technology at different time points during the experiments (mean with 95% CI) in the same animals as described in the legend to Fig. 1. Statistical significance is given for the difference between the positive control and the treatment group

In the positive control group, IFN-γ increased from <lower detection limit (LDL) at T0 to mean 16 (±7.5) pg/mL at T120 and 14 (±6.4) pg/mL at T180 (Fig. 2). In the treatment group, IFN-γ increased from <LDL at T0 to mean 2 (±2.3) pg/mL at T120 and 8 (±5.3) pg/mL at T180. The difference between the groups was statistically significant (*p* = 0.0001), and the area under the curve (AUC) was 75% lower in the treatment group than in the positive control group. No increase was seen in sham animals.

#### *TNF:*

In the positive control group, TNF increased from <LDL at T0 to mean 44 (±15.1) ng/mL at T120 (Fig. 2). Thereafter, the level declined to mean 13 (±4) ng/mL at T180. In the treatment group, TNF increased from <LDL to a plateau of mean 17 (±6.4 and ±10) ng/mL at T120 and T180. The difference between the groups was statistically significant (*p* = 0.01), and the AUC was 50% lower in the treatment group than in the positive control group. No increase was seen in sham animals.

#### *IL-1β:*

In the positive control group, IL-1β increased from <LDL at T0 to mean 0.1 (±0.05) ng/mL at T120 and 0.9 (±0.5) ng/mL at T180 (Fig. 2). In the treatment group, IL-1β increased from <LDL at T0 to mean 0.08 (±0.01) ng/mL at T120 and 0.6 (±0.3) ng/mL at T240. The AUC was 30% lower in the treatment group than in the positive control group but the difference between the groups was not significant (*p* = 0.2). No increase was seen in sham animals.

#### *IL-6:*

In the positive control group, IL-6 increased from <LDL at T0 to mean 0.8 (±0.3) ng/mL at T120 and 2.6 (±0.9) ng/mL at T180 (Fig. 2). In the treatment group, IL-6 increased from <LDL at T0 to mean 0.5 (±0.2) ng/mL at T120 and 2.6 (±1.1) ng/mL at T180. The AUC was 15% lower in the treatment group than in the positive control group, but the difference between the groups was not significant (*p* = 0.6). No increase was seen in sham animals.

#### *IL-8*:

In the positive control group, IL-8 increased from <LDL at T0 to mean 1.8 (±1) ng/mL at T120 and 4.5 (±1.6) ng/mL at T180 (Fig. 2). In the treatment group, IL-8 increased from <LDL at T0 to 0.4 (±0.2) ng/mL at T120 and 3.4 (±1.7) ng/mL at T180. The difference between the groups was statistically significant (*p* = 0.03), and the AUC was 50% lower in the treatment group than the positive control group. No increase was seen in sham animals. One animal of the treatment group showed excessively increased IL-8 level at T180 right before it died (83 ng/mL). This value was 33 SD higher than the mean value of the rest of the treatment group. Thus, it was regarded as an outlier and excluded from statistical analysis.

#### *IL-10:*

In the positive control group, IL-10 increased from mean 0.05 (±0.01) ng/mL at T0 to 4.6 (±0.8) ng/mL at T120 and 6.6 (±2.3) ng/mL at T180 (Fig. 2). In the treatment group, IL-10 increased from mean 0.1 (±0.01) to 2.8 (±0.4) ng/mL at T120 and 3.6 (±0.4) ng/mL at T180. The difference between the groups was statistically significant (*p* = 0.04), and the AUC was 40% lower in the treatment group than the positive control group. No increase was seen in sham animals.

#### *IL-12p40:*

In the positive control group, IL-12p40 increased from mean 0.5 (±0.01) ng/mL at T0 to 1.8 (±0.3) ng/mL at T120 and 10.0 (±2.5) ng/mL at T180 (Fig. 2). In the treatment group, IL-12p40 increased from mean 0.4 (±0.01) ng/mL at T0 to 0.7 (±0.1) ng/mL at T120 and 5.4 (±1.2) ng/mL at T180. The difference between the group was statistically significant (*p* = 0.03), and the AUC was 50% lower in the treatment group than the positive control group. No increase was seen in sham animals.

IFN-α and IL-4 were not increased in any of the groups (data not shown).

### Granulocyte activation

#### *wCD11R3:*

**Fig. 3:**
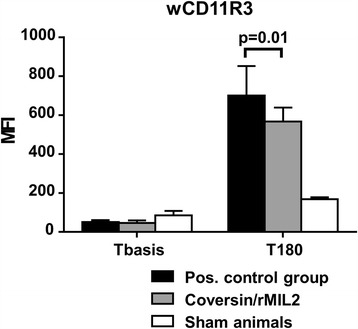
wCD11R3 expression on the surface of granulocytes (mean with 95% CI) was measured by flow cytometry at baseline (Tbasis) and at the end of the experiment (T180) in the same animals as described in the legend to Fig. 1. Statistical significance is given for the difference in MFI between the positive control and the treatment group at T180

In the positive control group, wCD11R3 increased from mean MFI 51 (±9) at T0 to MFI 700 (±150) at T180, whereas in the treatment group wCD11R3 increased from mean MFI 46 (±14) at T0 to MFI 568 (±68) at T180 (Fig. 3). The difference between the two groups was significant (*p* = 0.01).

## Discussion

Fulminant meningococcal sepsis is associated with a massive, rapid, and harmful inflammatory activation that is rarely seen in other conditions. CD14-mediated activation of the Toll-like receptor system and activation of complement are the two best characterized parts of the innate immune system participating in this inflammatory response. Previously, we have performed extensive studies on the effect of blocking CD14 and complement individually and together in both in vitro and in vivo inflammatory models [10, 18, 32]. The results from these studies have clearly demonstrated attenuation of the inflammatory response when these two parts of the innate immune system are blocked together, compared to separate blocking. Thus, based on the previous studies, and not least in order to limit the total number of animals used, this study was designed to investigate only the effect of combined CD14 and complement inhibition in meningococcal sepsis. This combined blocking of C5 and CD14 significantly and efficiently attenuated the inflammatory response induced by *N. meningitidis* in vivo*.*

Sustained high-grade activation of complement continuing until death despite optimal antibiotic and conventional supportive treatment is a characteristic for non-surviving patients with fulminant meningococcal sepsis [33]. Persistent high levels of complement-derived inflammatory molecules may contribute to the development of the systemic inflammatory response syndrome (SIRS). The potent anaphylatoxin C5a is considered the most important of these mediators, and thus blocking complement at the level of C5 is an interesting approach in fulminant meningococcal sepsis. Abolished delivery of C5a will attenuate the inflammatory response, while simultaneous administration of antibiotic treatment ensures killing of meningococci despite the lack of C5b-9 lysis. Also, activation of complement C3 is maintained when C5 is blocked, leaving C3-mediated opsonophagocytosis active for clearance of bacteria.

In the present study, we reproduced the systemic complement activation seen in patients with fulminant meningococcal sepsis. For the first time, it is demonstrated that this complement activation can be efficiently abolished by a C5 inhibitor in vivo, suggesting that such treatment can have a potential role as adjuvant treatment in patients with fulminant meningococcal sepsis.

Combined treatment with C5 and CD14 inhibition was applied to tackle two important inflammatory mechanisms at the earliest possible stage during immune recognition of meningococci. The goal of inhibiting the innate immune response in cases of fulminant meningococcal sepsis should be to interfere with the initial pro-inflammatory signals. This is to avoid further escalation of the cytokine response and prevent development of “point of no return.” Then it is impossible to reverse the highly disturbed pathophysiology of this condition. Presently, the most effective method to downregulate plasma cytokine level in patients with fulminant meningococcal sepsis is appropriate antibiotic treatment. The bacterial load as determined by number of *N. meningitidis* DNA molecules per milliliter plasma is reduced by 50% within 3 to 4 h after initiation of antibiotic treatment [34]. In parallel, key inflammatory cytokines and chemokines are reduced by 50% in plasma within 1 to 3 h [35, 36]. Even though the present study simulated a situation without antibiotics and escalating number of meningococci, the combined blocking of C5 and CD14 alone obtained a substantial attenuation of the release of several key cytokines, i.e., INF-γ, TNF, IL-8, IL-10, and IL-12p40. Our present experiments suggest that combined C5 and CD14 inhibition might reduce the cytokine production in patients and add to the effect of antibiotics per se.

The inhibitory effects on TNF and IL-10 by the combined treatment are of particular interest. TNF was efficiently but transiently inhibited in the initial phase, in a way that abolished the high peek in TNF concentration otherwise seen in the positive control group as well as in previous studies [18, 21]. TNF is a strong inductor of the acute phase response in SIRS and is the most extensively studied cytokine in sepsis [37]. High levels of TNF in the initial phase of sepsis have been associated with early hemodynamic deterioration [37, 38]. Thus, attenuated increase of TNF in the initial phase of sepsis development may be particularly beneficial. There is evidence that with progression of sepsis anti-inflammatory mechanisms become increasingly important [37, 39, 40]. IL-10 is the most potent anti-inflammatory cytokine, being substantially increased in meningococcal sepsis [41–43]. Sustained high levels of IL-10, and in particular sustained high IL-10/TNF ratio have been demonstrated to be associated with high mortality in meningococcal sepsis as well as in other infectious diseases [39, 44, 45]. IL-10 is responsible for extensive changes in the function of monocytes in meningococcal sepsis [43, 46]. Based on these observations, we assume that it may be beneficial to decrease the level of IL-10 in meningococcal sepsis, like we obtained with the combined treatment in this study. Furthermore, we suggest that the balance between TNF and IL-10 may be altered in a favorable way in the initial as well as later stages of sepsis development by the combined treatment. This may be in contrast to isolated inhibition of pro-inflammatory cytokines like TNF and IL-1β, which has been tried without success [40]. Given the substantial amounts of cytokines released “downstream” of the innate immune recognition phase, it is perhaps not surprising that inhibition of only one of these does not have an effect on the clinical course of the fulminant form of the disease. Thus, “upstream” inhibition of bottle-neck molecules of the main pattern recognition systems, like complement and CD14/TLRs, is a rational approach for broad-acting attenuation of the inflammatory response induced by meningococci.

Further, we suggest that the efficient inhibition of the chemokine IL-8 that we obtained initially in this study can be beneficial with respect to the prognosis. High levels of IL-8 have been described to predict fatal sepsis [47–50]. IL-8 attracts leukocytes to the site of inflammation, and high levels of IL-8 are likely to contribute to the profound leukopenia characteristic of severe meningococcal sepsis.

The significantly reduced expression of granulocyte wCD11R3 (the porcine ortholog to human CD11b) in the treatment group implies that combined treatment influenced white blood cell function not only by decreasing cytokine release but also by decreasing expression of surface molecules. In complex with CD18, wCD11R3 constitutes complement receptor 3 (CR3), which is active in phagocytosis of bacteria. Phagocytosis per se is obviously beneficial in meningococcal disease in order to clear bacteria from the circulation. However, it does not need to be as efficient in the presence of antibiotics, and a limited decrease of phagocytosis may possibly be beneficial due to less induced oxidative burst, which otherwise may be harmful to surrounding tissue [32].

## Conclusions

We suggest that the course of meningococcal sepsis observed in piglets in the present study represents a realistic model of the situation in patients with fulminant meningococcal sepsis. We demonstrate that efficient complement inhibition can be obtained in this model. Furthermore, we find that combined inhibition of the two key innate immune systems, complement with the central component C5 and the TLRs with the co-receptor CD14, can modulate the inflammatory response in fulminant meningococcal sepsis, provided the C5 and CD14 inhibition is initiated early. Modulation of this inflammatory response may be of potential benefit in patients with meningococcal sepsis.
